# Spatial expression patterns of autophagy genes in the eye lens and induction of autophagy in lens cells

**Published:** 2012-06-30

**Authors:** Lisa Ann Brennan, Wanda Lee Kantorow, Daniel Chauss, Rebecca McGreal, Shuying He, Lyndzie Mattucci, Jianning Wei, S. Amer Riazuddin, Ales Cvekl, J. Fielding Hejtmancik, Marc Kantorow

**Affiliations:** 1 Department of Biomedical Science, Florida Atlantic University, Boca Raton, FL; 2 Departments of Ophthalmology and Visual Sciences and Genetics, Albert Einstein College of Medicine, Bronx, NY; 3 The Wilmer Eye Institute, Johns Hopkins University, School of Medicine, Baltimore, MD; 4 OGVFB, NEI, NIH, Bethesda, MD

## Abstract

**Purpose:**

Mutation of the autophagy gene *FYVE* (named after the four cysteine-rich proteins: Fab 1 [yeast orthologue of PIKfyve], YOTB, Vac 1 [vesicle transport protein], and EEA1) and coiled coil containing 1 (*fyco1)* causes human cataract suggesting a role for autophagy in lens function. Here, we analyzed the range and spatial expression patterns of lens autophagy genes and we evaluated whether autophagy could be induced in lens cells exposed to stress.

**Methods:**

Autophagy gene expression levels and their spatial distribution patterns were evaluated between microdissected human lens epithelium and fibers at the mRNA and protein levels by microarray data analysis, real-time PCR and western blot analysis. Selected autophagy protein spatial expression patterns were also examined in newborn mouse lenses by immunohistochemistry. The autophagosomal content of cultured human lens epithelial cells was determined by counting the number of microtubule-associated protein 1 light chain 3B (LC3B)-positive puncta in cells cultured in the presence or absence of serum.

**Results:**

A total of 42 autophagy genes were detected as being expressed by human lens epithelium and fibers. The autophagosomal markers LC3B and FYCO1 were detected throughout the newborn mouse lens. Consistently, the autophagy active form of LC3B (LC3B II) was detected in microdissected human lens fibers. An increased number of LC3B-positive puncta was detected in cultured lens cells upon serum starvation suggesting induction of autophagy in lens cells under stress conditions.

**Conclusions:**

The data provide evidence that autophagy is an important component for the function of lens epithelial and fiber cells. The data are consistent with the notion that disruption of lens autophagy through mutation or inactivation of specific autophagy proteins could lead to loss of lens resistance to stress and/or loss of lens differentiation resulting in cataract formation.

## Introduction

The eye lens is an avascular encapsulated organ whose function is to focus light on to the retina where visual information is translated into nerve signals and ultimately perception of images by the visual cortex of the brain. Disruption of lens transparency as a consequence of developmental defect or damage to lens cells and their components causes cataract formation [1–3] which is opacity of the eye lens and is the leading cause of world blindness [4]. The lens contains only a layer of cuboidal undifferentiated epithelial cells on top of a layer of elongated and differentiated lens fiber cells [5]. Lens fiber cells and their components are not renewed and must remain intact to maintain lens transparency throughout the life of the organism. The lens is exposed to photo-oxidative and other environmental damage making the lens an excellent model to study how environmental and oxidative stresses cause cellular and intercellular damage associated with aging [3,6–8]. The lens grows throughout the life of the organism through the slow differentiation of lens epithelial cells into lens fiber cells, which also makes the lens an excellent model for understanding those events important for cellular differentiation and longevity. Damage to the lens and its protein components results in cataract formation [3,7–9]. The differentiation of lens epithelial cells into mature lens fiber cells is accompanied by the degradation of mitochondria, nuclei and other organelles [5]. Failure of lens cells to complete the differentiation process can result in aberrant lens cell structure and inherited cataract formation [10]. Cataract also occurs as a consequence of environmental damage to lens cells and their components as a result of inadequate functioning of protective and repair mechanisms [3,9].

One intriguing system that may be important for lens differentiation and resistance to environmental damage is macroautophagy (hereafter termed autophagy) which operates in the degradation and recycling of damaged organelles and proteins in many other tissues [11]. Autophagy is characterized by the formation of autophagosomes which are double-membrane structures that engulf damaged cellular components and traffic them to lysosomes where the components are degraded/recycled [11–13]. Autophagy has been shown to be important for development [14], aging [15,16], and neurodegeneration [17,18]. Mitophagy, a specialized form of autophagy is a selective process whereby damaged mitochondria are specifically degraded in cells [19,20]. Both autophagy and mitophagy could be important for the removal of damaged lens cells, proteins and organelles. Loss of autophagy and/or mitophagy could, therefore, result in cataract formation.

To date, autophagy has not been extensively studied in the lens. Although autophagic vesicles containing mitochondria and other components were detected by electron microscopy as early as 1984 [21,22] the only paper to address autophagy in the lens reported that, deletion of the autophagy induction gene autophagy related 5 (*ATG5*) did not disrupt lens fiber cell differentiation despite the occurrence of autophagy in lens cells [23]. Since autophagy is now known to involve ATG5-dependent and ATG5-independent pathways [24] the conclusion that autophagy is not required for lens cell differentiation is no longer supported by the literature.

We have recently demonstrated that mutations in the gene encoding FYVE (named after the four cysteine-rich proteins: Fab 1 [yeast orthologue of PIKfyve], YOTB, Vac 1 [vesicle transport protein], and EEA1) and coiled coil containing 1 (*FYCO1)* are associated with the inheritance of autosomal recessive human cataract [25] suggesting that autophagy is likely required for the maintenance of lens transparency. FYCO1 is a FYVE and coiled-coil domain containing protein that has been demonstrated to be important for transport of autophagosomes to lysosomes where autophagosomal cargo is degraded [26]. In lens cells, FYCO1 was demonstrated to co-localize with the autophagosomal marker microtubule-associated protein 1 light chain 3B (LC3B) and lysosomes [25]. Thus FYCO1 could be important for removal of organelles during lens fiber cell differentiation and/or removal of damaged lens proteins.

Based on this study, and the potential importance of autophagy in lens function, we hypothesized that autophagy is important for lens function and resistance to cataract formation. To test this hypothesis, we analyzed the spectrum and range of autophagy genes expressed in microdissected human lens epithelium and fibers, we confirmed the mRNA and protein expression levels of functional subsets of autophagy genes in these lens sub-regions, we examined the spatial expression patterns of the autophagosomal marker LC3B and FYCO1 in whole mouse eyes and we monitored numbers of LC3B positive puncta in cultured human lens cells exposed to serum starvation which is a well characterized autophagy inducer in multiple cell types [12].

Our analysis revealed the lens epithelium and fiber expression of 14 genes involved in the induction of autophagy, eight genes involved in expansion and closure of autophagosomes, six genes involved in autophagosome fusion to lysosomes and eight genes involved in specific autophagy sub-pathways including mitophagy and chaperone-mediated autophagy. Consistent with a function for these genes in lens cells, the autophagosomal marker LC3B and the autophagy protein FYCO1 were detected to be present throughout the newborn mouse lens by immunohistochemistry and the active form of LC3B (LC3B II) was detected in microdissected human lens fibers, suggesting that autophagy is an actively occurring process in the lens fibers. Consistently, increased numbers of LC3B-positive puncta were detected in serum-starved lens epithelial cells relative to untreated cells suggesting that autophagy is an important response of the lens to environmental stress. Collectively, these data provide evidence that autophagy is required for lens function and that its disruption could lead to loss of lens stress resistance, loss of lens cell differentiation and ultimately cataract formation.

## Methods

### Gene expression analysis of specific autophagy transcripts

The levels of autophagy transcripts were analyzed from Affymetrix (U133A) microarray (Affymetrix, Santa Clara, CA) gene signature intensities detected upon hybridization with reverse transcribed and fragmented total lens RNA isolated from pooled microdissected human lens epithelium (7–9 mm central) and fibers (rest of lens; average age 57.8, age range 47–69). These data were previously reported in part [27]. Raw affymetrix chip data were normalized between lens epithelium and fiber cell populations using the housekeeping genes *GAPDH* (glyceraldehyde-3-phophate dehydrogenase), *PGK* (phosphoglycerate kinase), and *TRP* (trieosphate isomerase) as standards. Selected autophagy transcripts were further evaluated by semi-quantitative real-time PCR (RT–PCR) using the SuperScript® III one-step RT–PCR system with Platinum Taq polymerase (Invitrogen, Carlsbad, CA) according to the manufacturer’s instructions and *GAPDH* as control. We assayed 50–100 ng of total RNA from human microdissected lens tissue. RNA was isolated from microdissected human lens epithelium and fiber cells as previously described [28] using the Total RNA kit (Ambion, Woodland, Tx) according to the manufacturer’s instructions. A summary of primers used is provided in Table 1. PCR cycle numbers were chosen to be linear at the indicated amounts of RNA and cycle numbers (Table 1).

**Table 1:** Oligonucleotide primers used in semi-quantitative RT–PCR.

Gene	Forward primer	Reverse primer	NCBI#	Cycles	ng RNA	Annealing temp
*beclin 1*	CGGGAAGTCGCTGAAGACAG	CCATCCTGGCGAGGAGTTTC	NM_003766.3	30	100	55
*atg14*	GAGCGGCGATTTCGTCTACT	CTGAAGACACATCTGCGGGG	NM_014924.4	35	50	55
*mtor*	TTCTGGTGCGACACCGAATC	CATCGGGTTGTAGGCCTGTG	NM_004958.3	30	100	55
*rb1cc1/fip200*	GGAGCTTGTGCACCTGAACT	GAAGCACCCTCACCTGGTTTG	NM_014781.4	35	50	55
*ralb*	GCTCGTCGTGGGAAACAAGT	TGACAAAGCAGCCCTTCCAC	NM_002881.2	35	50	55
*atg4a*	GAGTAAGGGCACCTCTGCCTA	GTTCATTCGCTGTGGGGACT	NM_052936.3	35	50	55
*atg12*	GAGGTCTGTAGTCGCGGAGA	TGGATGGTTCGTGTTCGCTC	NM_004707.3	30	100	55
*map1lc3b /atg8*	AAGTGGCTATCGCCAGAGTCG	CTGAGATTGGTGTGGAGACGC	NM_022818.4	25	100	55
*rab7*	GACACAGCAGGACAGGAACG	TTGTACAGCTCCACCTCCGT	NM_004637.5	25	100	55
*fyco1*	GAAGCTGAAGGCCACCCAAG	GGGCATCTGACTTCTGCCAG	NM_024513.3	35	50	55
*bnip3l/nix*	ACTCGGCTTGTTGTGTTGCT	TCCCTGCTGGTGTGCATTTC	NM_004331.2	25	100	58
*pink1*	TCTGCAGTCCTCTGCTCACA	GCTCATCCGTCACTTTCGCT	NM_032409.2	35	50	55
*p62*	CTCACCGTGAAGGCCTACCT	TAGCGGGTTCCTACCACAGG	NM_003900.4	35	50	55
*GAPDH*	CCACCCATGGCAAATTCCATGGCA	TCTAGACGGCAGGTCAGGTCCACC	NM_003900.4	35	50	60

The corresponding levels of autophagy proteins were further analyzed by western analysis. Protein samples were mixed with 2× Laemmli sample buffer (0.5 M Tris-HCl, pH 6.8, Glycerol 10% [w/v], SDS 0.1% [w/v], 0.0025% Bromophenol Blue, and 5% 2-Mercaptoethanol) at a 1:1 volume ratio and heated at 100 °C for 5 min. Samples were separated by electrophoresis on 8%, 10%, and 15% sodium dodecyl sulfate-polyacrylamide gels where appropriate at room temperature using a Bio-Rad mini Protean® vertical electrophoresis system (Bio-Rad, Hercules, CA). Proteins were transferred onto Hybond™ ECL™ nitrocellulose membrane (GE Healthcare, Buckinghamshire, UK) using a Bio-Rad mini Trans Blot® electrophoresis system (Bio-Rad) for 1.5 h at 100 V. Following transfer immunoblots were rinsed in phosphate buffered saline (PBS) pH 7.2 for 2 min. Immunoblots were then blocked in 5% milk in Tris Buffered Saline with Tween (TBST; 5% fat-free dry milk, 0.1% Tween-20, 150 mM NaCl, and 50 mM Tris at pH 7.5) for 1 h before incubation with the appropriate primary antibody diluted in 5% milk TBST (anti-LC3B antibody [Abcam, Cambridge, MA] 1:1,000, anti-RB1CC1/FIP200 [Bethyl Labs, Montgomery, TX] 1:1,000, anti-FYCO1 [Bethyl laboratories] 1:1,000 and anti-BNIP3L/NIX [Enzo Life Sciences, Plymouth Meeting, PA] 1:2,000). Blots were washed in TBST and incubated for 1 h with 1:5,000 DyLight goat anti rabbit 800 conjugated secondary antibody (Thermo Scientific, Rockford, IL) followed by rinsing in PBS pH 7.2 for 2 min. Immunoblots were imaged for 2 min on the Odyssey Imaging System (LI-COR Biosciences, Lincoln, Nebraska).

### Spatial localization of LC3B and FYCO1 proteins in mouse lens

Animal husbandry and experiments were conducted in accordance with the approved protocol of Animal Institute Committee (Albert Einstein College of Medicine, NY) and the Association of Research in Vision and Ophthalmology (ARVO) Statement for the Use of Animals in Ophthalmic and Vision Research. Noon of the day that the vaginal plug was observed was considered as E0.5 of embryogenesis.

Pregnant female mice were euthanized by CO_2_ and sacrificed following standard procedure. Mouse embryos were dissected and then fixed in 10% neutral buffered paraformaldehyde overnight at 4 °C before paraffin embedding. Serial sections were cut in 5 μm thick sections through the mid-section of the lens. Immunohistological staining was performed following standard procedures described below. Antigen retrieval was performed to unmask the paraffin embedded tissues before antibody incubation.

Whole mouse head sections were processed from a postnatal day 1 (P1) mouse, and LC3B and FYCO1 proteins were visualized by immunohistochemistry using the ImmPRESS Reagent kit according to the manufacturer’s instructions (Cat no. MP-7401; Vector Laboratories, Burlingame, CA). Briefly, tissues were deparafinized and hydrated using xylene and ethanol gradients and then rinsed in tap water for 5 min. The sections were blocked with 2.5% horse serum for 1 h. Primary FYCO1 (Cat no. A302–796A; rabbit polyclonal; Bethyl Labs) and LC3B antibodies (rabbit polyclonal; Sigma-Aldrich, St Louis, MO) were both diluted in 2.5% horse serum at 1:250, added to the sections and incubated overnight at 4 °C. The sections were washed in phosphate buffered saline contiatween 20 (PBS-T) for 5 min and incubated with the ImmPRESS reagent (Cat no. MP-7401; anti-rabbit immunoglobulin peroxidase, Vector Laboratories) at room temperature for 30 min according to the manufacturer’s instructions. The sections were washed again in PBS-T and incubated with ImmPACT DAB Peroxidase Substrate (Cat no. SK-4105; Vector Laboratories) for 4 min at room temperature. For the sections that were counterstained, Vector’s Hematoxylin QS (Cat No H-3404) was used according to the manufacturer’s instructions. Tissue sections were incubated with hematoxylin counterstain for 30 s at room temperature and dipped in tap water for 10 s to remove excess stain. Sections were cleared and mounted with VectaMount Permanent Mounting Medium (Cat no. H-5000; Vector Laboratories). Identical procedures were performed using only rabbit secondary antibody as a control. Sections were visualized using an Olympus Provis AX70 (Olympus, Center Valley, PA) fluorescent microscope and images captured using Magnafire software (Optronics, Goleta, CA).

### Lens cell culture

A human lens epithelial cell line (HLEB3) [29] (a gift from Dr. Majorie Lou, University of Nebraska-Lincoln, Lincoln, NB) was grown and cultured in Dulbecco Modified Eagle Medium (Invitrogen, Carlsbad, CA) supplemented with 15% fetal bovine serum (Invitrogen), gentamicin (50 units/ml; Invitrogen), penicillin-streptomycin antibiotic mix (50 units/ml; Invitrogen), and amphotericin B (1.25 µg/ml; Invitrogen) at 37 °C in the presence of 5% CO_2_. For induction of autophagy by serum starvation HLEB3 lens cells were plated in 24 well plates at a density of 50,000 cells per well overnight. For serum starvation, HLEB3 cells were transferred to serum-free media with or without addition of 50 µM chloroquine, an autophagy inhibitor that prevents autophagosome fusion with lysosomes [30], and assessed for autophagy at 24 h post treatment by staining with an LC3B specific antibody and fluorescent confocal microscopy as described below.

### LC3B accumulation assays

HLEB3 lens cells were plated onto coverslips and treated as described above for induction of autophagy using serum starvation. Immunofluoresence staining was conducted by fixing cells with 3.7% formaldehyde in PBS, blocking with 1% BSA and permeabilizing with 0.25% TritonX-100 in PBS. Following permeabilization, a rabbit polyclonal anti-LC3B (Sigma-Aldrich) at 1:1,000 was incubated overnight at 4 °C. Cells were washed three times with PBS, and subsequently incubated with Alexa Fluor 488 goat anti-rabbit secondary (Invitrogen) for 1 h at room temperature at a 1:2,000 dilution. HLEB3 cells were washed three times with PBS and the nucleus counterstained using 300 nM DAPI (Invitrogen) for 2 min. Cells were washed three times with PBS and mounted onto glass slides using ProLong Gold antiFade reagent (Invitrogen). Immunofluoresence staining was visualized with a Zeiss LSM 700 Confocal microscope (Zeiss, Thronwood, NY). LC3B puncta were quantified in at least 50 cells per treatment using the AxioVision 4 software (Zeiss) by manual visual selection of “events” as described below and the mean and standard deviation calculated. Fully rounded intense green staining of LC3B was counted as a single puncta or “event” representing an autophagosome; diffuse staining is believed to be cytoplasmic LC3 I and was not counted as puncta. Data presented is representative of 3 independent experiments. Differences between treatments and controls were determined using Tukey's test following one-way ANOVA. A p-value less than 0.001 was considered statistically significant.

## Results

### Repertoire of autophagy genes expressed by the human lens

**Figure 1:**
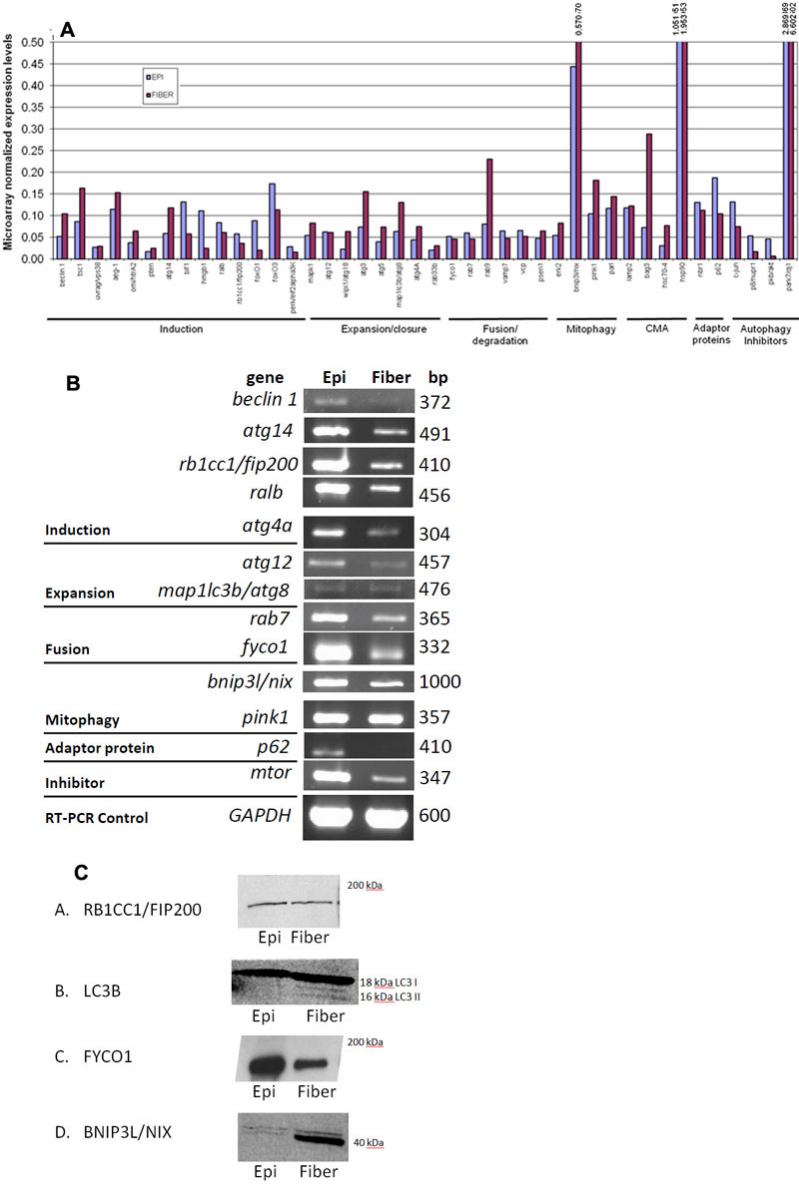
Expression of autophagy genes in lens epithelium and fibers. **A**: Histogram representation of microarray gene expression data. Data were normalized to the levels of *GAPDH* (glyceraldehyde-3-phophate dehydrogenase), *PGK* (phosphoglycerate kinase), and *TRP* (trieosphate isomerase). **B**: Autophagy gene expression in separately isolated human lens epithelium and fiber cells by semi-quantitative RT–PCR. **C**: Autophagy protein levels of indicated proteins in microdissected human lens epithelium and fiber cells.

Autophagy-associated gene expression levels were compared between human microdissected lens epithelium (7–9 mm central) and remaining fiber cells through analysis of microarray, semi-quantitative RT–PCR and western blot data. Autophagy-associated transcript levels were first determined through analysis of previously obtained microarray data [27] from 34 pooled human lens epithelium and fiber cell samples (average age 57.8, age range 47–69) analyzed on affymetrix U133A chips. Autophagy gene expression levels were normalized between lens epithelium and fiber cell samples relative to the housekeeping genes *GAPDH*, *PGK*, and *TRP*. This analysis identified the measurable expression in lens epithelium and lens fibers of 42 autophagy-associated genes including autophagy adaptor proteins (proteins involved in linking autophagy components and processes) and autophagy inhibitors. The data are shown in Figure 1A and a summary of the identified genes and their autophagy functions with references is shown in Table 2.

**Table 2:** Identified autophagy genes and their functions.

Induction	Role in Autophagy	Reference
Beclin 1	Member of PtdIns 3-kinase complex, involved in activation of macroautophagy	[31]
TSC1	Acts as a gtpase-activating protein for Rheb, thus inhibiting TOR	[32]
UVRAG	Member of PtdIns 3-kinase complex, regulates macroautophagy	[33]
AEG1	Gene encodes oncogenic protein that induces macroautophagy independent of Beclin-1 and PtdIns 3-kinase	[34]
Omi/HtrA2	Degrades the Bcl-2 family-related protein Ha × −1 to allow macroautophagy induction	[35]
Pten	Dephosphorylates PdtIns(3,4,5)P3 inhibiting PDK1 and PKB/Akt activity	[36,37]
Atg14	Component of PtdIns 3-kinase complex, targets this complex toward autophagic machinery	[38]
Bif-1	Interacts with Beclin 1 via UVRAG and is required for macroautophagy	[39]
HMGB1	Binds beclin-1 to displace Bcl-2 inhibiting apoptosis and promoting macroautophagy	[40]
RalB	Activation of phagophore assembly through ULK1-Beclin1-Vps34 complex assembly and Exo84 interaction	[41]
RB1CC1/FIP200	Component of Ulk1 complex, required for phagophore formation, phosphorylation of Ulk1/2	[42]
FoxO1	Regulates macroautophagy independent of transcriptional control	[43]
FoxO3	Stimulates macroautophagy through transcriptional control of autophagy genes	[44]
PERK/eif2α3K	Phosphorylated due to ER stress which induces LC3 conversion and macroautophagy	[45,46]
**Expansion/Closure**		
MAPK1	MAPK/ERK regulates the maturation of autophagosomes	[47]
Atg12	Ubiquitin-like protein, conjugates Atg5, member of ATG12–5–16 complex, essential for Map1LC3B/Atg8 activation, involved in mitochondrial homeostasis	[48,49]
WIPI1/Atg18	Binds PI3P by WD40 β-propeller domain, involved in retrograde movement of Atg9	[50,51]
Atg3	E2 ubiquitin ligase, conjugates PE to Map1LC3B after Atg7 processing of c-terminus of cleaved Map1LC3B/Atg8, can be conjugated to Atg12	[49,52]
Atg5	Contains ubiquitin-folds, member of ATG12–5–16 complex	[48]
Map1LC3b/Atg8	Atg8 homolog, involved in autophagosome biogenesis and cargo recruitment to autophagosomes, marker of autophagosomes	[53–55]
Atg4a	Cysteine Protease of Yeast Atg8 homologs, required for Map1LC3B /Atg8 activation, able to deconjugate PE of processed Map1LC3B	[55,56]
Rab33B	Binds Atg16L1, involved in autophagosome maturation by regulation of autophagosome to lysosome fusion, OATL1 binding partner	[57,58]
**Fusion/Degradation**		
FYCO1	Rab7 effector, binds Map1LC3B and phosphatidylinositol-3-phosphate, coordinates plus-end directed autophagosome transport	[26,59]
Rab7	Transport of early to late endosomes, docking protein for amphisome to lysosome fusion	[26,60–62]
Rab9	Involved in trafficking from late endosomes to the trans-golgi, believed to be a key component of the ATG5/7 alternative macroautophagy pathway	[60,63]
VAMP7	SNARE protein, required for autophagosome formation, autophagosome maturation via facilitation of autophagosome to lysosome fusion	[64,65]
VCP	AAA+ ATPase, required for autophagosome maturation, mutations to vcp results in accumulation of ubiquitin-containing autophagosomes	[66,67]
PSEN1	Protease, part of the γ-secretase complex, involved in lysosomal degradation	[68]
**Mitophagy**		
ERK2	Localizes to the mitochondria, regulates mitophagy	[69]
BNIP3L/NIX	Bcl2 related, necessary for selective mitochondrial clearance	[70]
Pink1	Decreased MMP causes altered Pink1 processing, results in spanning of Pink1 across the outer mitochondrialmembrane, recruiting Parkin for mitophagy	[71]
PARL	Mitochondrial protease that regulates PINK1 localization and stability	[72]
**Chaperone Mediated Autophagy**		
Lamp2	Lysosomal membrane receptor for chaperone-mediated autophagy allowing translocation of substrates across the lysosomal membrane.	[73]
BAG3	Directs Hsp70 misfolded protein substrates to dynein targeting them to aggresomes for selective degradation	[74]
Hsc70–4	Aids in targeting of cytosolic proteins to the lysosome for degradation	[75]
hsp90	Assists in LAMP-2A stabilization of during its lateral mobility in the lysosomal membrane	[76]
**Adaptor Proteins**		
NBR1	Binds ubiquitinated proteins allowing degradation by macroautophagy	[77,78]
P62	Interacts with Atg8 via its LIR domain, adaptor for degradation of ubiquitin-labeled molecules	[78,79]
**Autophagy Inhibitors**		
mTOR	Serine/threonine kinase that controls cells growth and metabolism in response to nutrients, growth factors, cellular energy and stress	[80,81]
c-Jun	transcription factor, Inhibits mammalian macroautophagy induced by starvation	[82]
p8/Nupr1	Inhibits macroautophagy by repressing the transcriptional activity of FoxO3	[83]
PKB/Akt	Upstream regulator of mtor	[84]
PARK7/DJ1	Overxpression suppresses macroautophagy through the JNK pathway	[85]

Of the genes identified in both lens epithelial and lens fiber cells, 14 genes were involved in autophagy induction (Bcl-2 interacting myosin/moesin-like coiled coil protein 1 [*beclin 1*], tuberous sclerosis complex 1 [*tsc 1*], UV irradiation resistance-associated gene [*uvrag*], astrocyte-elevated gene-1 [*aeg-1*], high temperature requirement  factor A2 [*omi*/*htrA2*], phosphatase and tensin homolog [*pten*], autophagy related 14 [*atg14*], Bax-interacting factor 1 [*bif1*], high mobility group box 1 [*hmgb1*], v-ral simian leukemia viral oncogene homolog B [*ralB*], retinoblastoma 1 inducible coiled coil-1/focal adhesion kinase (FAK) family interacting protein of 200 kDa [*rb1cc1/fip200*], forkhead box O1 [*foxO1*], forkhead box O3 [*foxO3*], and PKR-like ER kinase/eukaryotic translation initiation factor 2-alpha kinase 3 * *[*perk*/*eif2alpha3k*]), eight genes were involved in expansion of autophagic vesicles (mitogen activated protein kinase 1 [*mapk1*], autophagy related 12 [*atg12*], WD repeat domain phosphoinositide-interacting protein 1/autophagy related 18 [*wipi1*/*atg18*], autophagy related 3 [*atg3*], *atg5*, microtubule-associated protein 1 light chain 3B/autophagy related 8 [*map1lc3b*/*atg8*], autophagy related 4a [*atg4a*], and the small GTP-binding protein* rab33b*), six were genes involved in autophagosome fusion to lysosomes (*fyco1*, two members of the ras oncogene family; *rab7* and *rab9*, vesicle-associated membrane protein 7 [*vamp7*], valosin-containing protein/p97 [*vcp*], and presenilin 1 [*psen1*]) and eight genes were involved in specific autophagy sub- pathways including mitophagy (extracellular signal-regulated kinase 2**[*erk2*], Bcl-2/adenovirus E1B 19-kDa interacting protein 3-like/NIP3-like protein X [*bnip3l*/*nix*], PTEN-induced kinase 1/PARK6 [*pink1*], and presenilin associated rhomboid-like [*parl*]) and chaperone mediated autophagy (lysosome-associated membrane protein type 2 [*lamp2*]*, *Bcl-2-associated athanogene**[*bag3*], heat shock cognate 70 kDa protein 4 [*hsc70-4*], and heat shock protein 90 [*hsp90*]; Figure 1A, Table 2).

Of these, the expression levels of 12 autophagy-associated genes were further analyzed by semi-quantitative RT–PCR using a separately prepared RNA sample isolated from a second population of microdissected human lens epithelium and fiber cells (n=5, average age 42.6, age range 15–60; Figure 1B). One additional gene, mammalian target of Rapamycin (*mtor*), which was present on the microarray but not definitively identified to be expressed, was also analyzed. These genes included the autophagy induction genes *beclin 1, atg14, fip200, ralb*, the autophagy expansion/closure genes *atg4a, atg12, map1lc3b/atg8,* the autophagy fusion/degradation genes *rab7, fyco1,* the mitophagy genes *nix/bnip3L, pink1* and the adaptor gene sequestosome 1 (*p62*). The data confirmed that all of the analyzed genes were expressed in human lens epithelium and fiber cells (Figure 1B). Differences in absolute levels of the genes between microarray and RT–PCR data are consistent with variability in populations of human lenses and differences in techniques used.

Since protein levels of lens autophagy genes could differ from mRNA levels, the protein levels of four selected autophagy genes were also examined in protein extracts isolated from a third pool of separately isolated human lens epithelium and fiber cells (n=34, average age 57.8, age range 47–69; Figure 1C). Consistent with their detection at the mRNA level, autophagy proteins RB1CC1/FIP200, LC3B, and FYCO1 and the mitophagy-associated protein BNIP3L/NIX were detected in both lens epithelium and lens fiber cells. Interestingly, three bands were detected for LC3B in the fiber cells. The 18 kDa band is consistent with the unprocessed cytoplasmic LC3B I [53] while one of the two smaller bands likely represents the activated LC3B – LC3B II at approximately 16 kDa [53,55]. Although we do not know the exact LC3B modification in these two lower molecular weight bands, cytoplasmic LC3B I is known to be cleaved and lipidated during activation leading to a 16 kDa molecular weight band (LC3B II). LC3B II is specifically inserted into the autophagosomal membrane and is, therefore, a classic marker for the presence of autophagosomes [12,55]. Collectively, these data suggest the presence of an autophagy function in both lens epithelium and lens fiber cells.

### Spatial localization of LC3B and FYCO1 in the mouse eye lens

**Figure 2:**
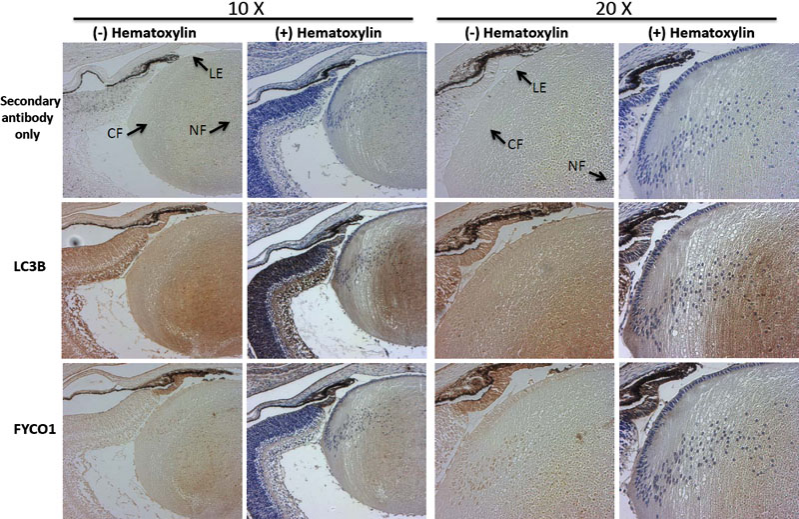
Spatial localization of LC3B and FYCO1 in whole mouse lenses. Immunostaining of LC3B and FYCO1 in postnatal day 1 mouse lens with LC3B-specific antibody and FYCO1-specific antibody. Secondary antibody alone is shown as control. Lens epithelium (LE), lens cortical fibers (CF) and nuclear fibers (NF) are indicated. Brown staining show positive antibody cross reactivity and blue hematoxylin staining is nuclear staining.

To determine the localization of some of the autophagy components in the lens and the rest of the eye, day one mouse lenses were sectioned and immunostained with LC3B- and FYCO1-specific antibodies (Figure 2). LC3B is a well characterized marker for autophagosomes [53–55,86,87], and FYCO1 is an autophagy protein associated with cataract [25]. The lens fiber cells consist of cortical fiber cells (CF) that are actively differentiating and still contain mitochondria and nuclei and nuclear fiber cells (NF) which lack nuclei and other organelles and are terminally differentiated. Since our RNA data (above) does not distinguish between cortical and nuclear fibers data using whole lens allows this distinction. The LC3B autophagosomal marker was detected throughout the lens in both lens epithelium and fiber cells although, interestingly, the highest level of LC3B immunoreactivity was detected in the lens nuclear fibers, indicating that autophagy may have played a role in lens fiber cell differentiation. Mouse lens sections were also immunostained with antibody specific to FYCO1 (Figure 2) since its mutation is associated with lens cataract formation [25]. FYCO1 was localized throughout the lens and exhibited a very similar staining pattern to LC3B (Figure 2). The data are consistent with our previous study which demonstrated co-localization of LC3B and FYCO1 in cultured human lens epithelial cells [25].

### Induction of autophagy in human lens epithelial cells by serum starvation

**Figure 3:**
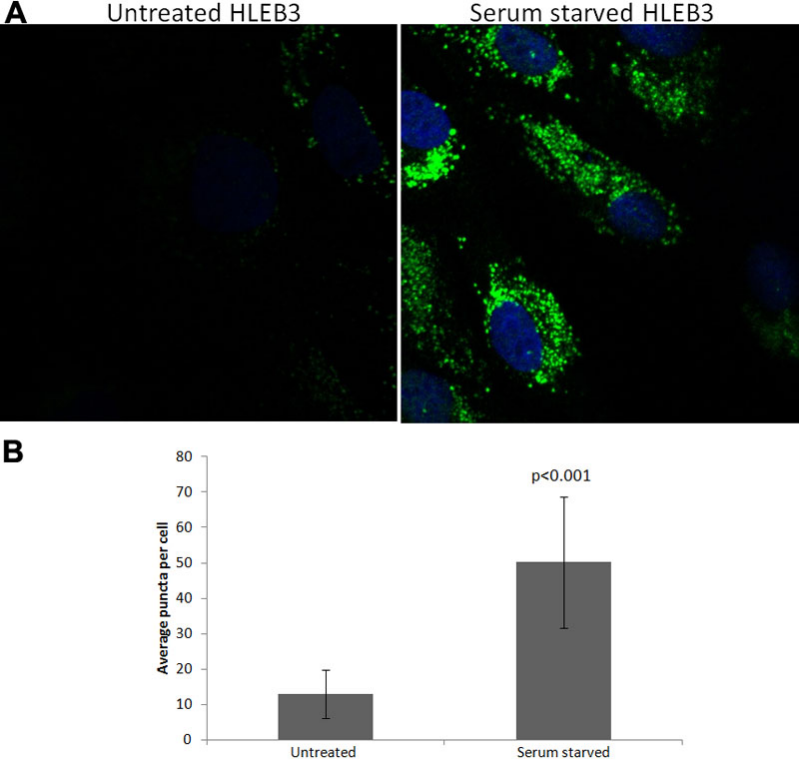
LC3B levels in serum starved human lens epithelial cells. **A**: LC3B levels in lens epithelial HLEB3 cells exposed to serum starvation and chloroquine (50 µM) detected by immunoflourescent confocal imaging (green). The nucleus is shown by DAPI staining (blue). **B**: Mean number of LC3B puncta are shown for each treatment (n=50 cells; error bars represent standard deviations). The data are statistically significant at p<0.001 by Tukey analysis.

The detection of expression of multiple autophagy genes in lens epithelium and fibers along with the presence of activated LC3B (LC3B II) in lens fibers suggests that autophagy is a functional process throughout the lens. Since autophagy is a response to stress in many tissues [12] we examined whether it could be induced in lens cells upon serum-starvation. We therefore attempted to detect increased levels of autophagosomes in lens cells by measuring increased numbers of LC3B-positive puncta in cultured human lens epithelial cells exposed to serum starvation and 50 µM chloroquine. Chloroquine is known to prevent autophagosomal fusion with lysosomes allowing visualization of accumulated LC3B II stained autophagosomes that would otherwise be turned over and the signal lost. Human HLEB3 epithelial cells were serum starved for 24 h and levels of LC3B monitored by fluorescent confocal microcopy. Quantitative analysis of LC3B-positive puncta revealed that LC3B-positive puncta numbers increased almost fourfold in lens epithelial cells upon serum starvation and chloroquine addition compared to cells incubated for the same time in complete media with identical chloroquine addition (Figure 3). Statistical analysis of LC3B-positive puncta numbers obtained by direct counting in 50 cells per treatment demonstrated significant differences (p<0.001) in LC3B positive puncta number between untreated control lens epithelial cells and lens epithelial cells exposed to serum-starvation.

## Discussion

Autophagy has been shown to be important for the development, differentiation and protection against oxidative stress in multiple tissues [14,88], however, to date its role in lens function has remained unexamined. The lens requires the removal of mitochondria and other organelles for fiber cell differentiation and requires multiple protective and repair systems to protect against damage. Since autophagy and mitophagy are critical for these functions in other tissues it is surprising that autophagy and mitophagy have not been explored for their likely role in lens differentiation, lens stress resistance and cataract formation. Recently, it was discovered that mutation of the autophagy gene *fyco1* causes human cataract [25], suggesting that autophagy is indeed important for lens differentiation and/or resistance to damage. Consistent with this hypothesis, FYCO1 co-localizes to lysosomes and autophagosomes in cultured lens epithelial cells [25].

As a first step toward examining the potential function of autophagy in the lens, we analyzed the expression levels of autophagy genes in microdissected human lens epithelium and fiber cells. Our analysis revealed the lens epithelium and fiber cell expression of 14 genes involved in autophagy induction, eight genes involved in expansion of autophagy vesicles, six genes involved in autophagosome fusion to lysosomes and eight genes involved in specific autophagy sub-pathways including mitophagy and chaperone mediated autophagy (Figure 1A). The data suggest that all components of the autophagy pathway, including those involved in induction, expansion/closure, and fusion degradation, as well as mitophagy and chaperone-mediated autophagy are present and likely functional in lens cells. Since both lens epithelium and fiber cells express these genes they are likely to have functions in both of these lens sub-components. The fiber-preferred expression of some of these genes also provides confidence that their presence in fiber cells is not an artifact of lens epithelial cell contamination or their persistence subsequent to lens cell differentiation. This interpretation is supported by the detection of high levels of LC3B and FYCO1 throughout the newborn mouse lens including lens fibers (Figure 2) and the detection of activated LC3B in the fiber cells by western analysis (Figure 1C). Consistent with an active autophagy pathway in lens cells we detected increased numbers of LC3B-positive puncta and therefore autophagosome number in lens cells exposed to serum starvation which is a well characterized autophagy inducer in a multitude of other studies and cell types (Figure 3). This observation also suggests that autophagy is a response of lens cells to exogenous stress that is likely involved in removing oxidized and aggregated proteins that are known to accumulate in lens cells upon oxidative and other stress conditions.

To date, few reports on the role of autophagy in lens function have been published. Matsui et al. [23] reported that the lenses of mice containing a homozygous knockout of the autophagy inducer ATG5 still developed organelle-free fiber cells, suggesting that this individual autophagy induction pathway was not required for organelle loss during lens cell differentiation. The data did, however, demonstrate that autophagy occurred in the embryonic mouse lens. Recently, Nishida et al. [24] discovered that an alternative, Atg5/7-independent, autophagy pathway operates in conjunction with the ATG5 pathway in non-lens cells to initiate autophagy. The lack of effect of ATG5 deletion on lens fiber cell organelle degradation suggests that the ATG5/7-independent pathway may operate to clear lens organelles during lens cell differentiation. Consistently, two members of the ATG5/7-independent pathway, Rab9 and beclin-1, were detected in both lens epithelium and lens fiber cells in the present study (Figure 1A). Recently Menko and Basu (Thomas Jefferson University, Philadelphia, PA, personal communication) reported autophagy was required for degradation of organelles during lens cell differentiation, providing even more evidence that autophagy is indeed involved in lens fiber cell organelle degradation.

In addition to the ATG5 mouse knockout, other knockout mice have been made for some of the genes we detected in the lens but unfortunately no lens or eye phenotype has been described for any of the mice. These include *map1lc3b* [89], *bnip3l/nix* [90,91], *beclin1* [92], *rb1cc1/fip200* [93], *bif-*1 [39], and *lamp2* [94]. Of these, *beclin 1* and *fip200* were embryonic lethal and *atg3* was neonatal lethal. *Lamp2* knockout mice were viable but exhibited increased mortality and *bif-1, map1LC3b* and *bnip3l/nix* were viable. Only two genes (apart from *fyco1)* that were identified in the present report have a previously reported lens function; these are *fox01* and *fox03* [95] which were implicated in lens oxidative stress resistance. Another autophagy gene, not detected in the lens in the present study, called ataxia telangiectasia mutated (*atm*), has also been reported to be important for lens resistance to cataract [96]. It is intriguing to speculate that the known autophagy functions of these genes could play a role in oxidative stress resistance by lens cells.

In summary, the present data provide evidence for a significant role for autophagy in lens function. Autophagy and mitophagy are likely important for lens cell removal of damaged proteins that could cause cataract upon their accumulation and the degradation of lens organelles during epithelial cell differentiation into fiber cells. Further studies on the role of autophagy in lens resistance to stress and lens cell differentiation are likely to provide additional insight into our understanding of lens development, maintenance and cataract formation.
